# Supplementary material for: Content-rich biological network constructed by mining PubMed abstracts
Source: BMC Bioinformatics. 2004 Oct 8;5:147. doi: 10.1186/1471-2105-5-147 (PMC528731; doi:10.1186/1471-2105-5-147)
Supplement: Additional File 5 — The original Chilibot query results of the term "long-term potentiation (LTP)" and 22 other terms, limiting the latest references analyzed to the years 1990, 1995, 2000, and 2004. [file 1471-2105-5-147-S5.bz2 › chilibotAdditionalFile5/ltp2004/html/PKC_TAU.html]

 


 **PKC** and **TAU** 
  
Found 64 abstracts in PubMed,  **30 abstracts were retrieved and analyzed**.  


---

 Search Google  |
 PDF files only 
|  EDU domain only 

---

**Interactive relationship** (e.g. stimulation, inhibition, etc)

- Glycogen synthase kinase 3 GSK 3beta phosphorylates  **tau**  and is inhibited via protein kinase C  **PKC** .  Ref: 11129110 J Neural Transm, 2000
- Significantly, specific inhibition of GSK 3 activity by LiCl prevents hyperphosphorylation of  **tau** , and spatial memory impairment resulting from PI3K and  **PKC**  inhibition.  Ref: 14713290 J Neurochem, 2003
- These epitopes were masked by higher  **PKC**  concentrations, suggesting the presence of multiple  **tau**  phosphorylation sites for which  **PKC**  exhibited differing affinities and or conformational alterations in  **tau**  induced by sequential  **PKC**  mediated phosphorylation.  Ref: 10081606 Cell Mol Neurobiol, 1999
- Site specific phospho  **tau**  immunoreactivity was increased in additive and synergistic manners by treatment of injected cells with TPA which activates  **PKC** , calcium ionophore which activates calcium dependent kinases, and wortmannin which inhibits PIP3 kinase.  Ref: 10081608 Cell Mol Neurobiol, 1999
- Protein kinase C  **PKC**  is known to increase non amyloidogenic alpha secretase cleavage of APP, producing secreted APP sAPPalpha, and glycogen synthase kinase GSK 3beta is known to increase  **tau**  phosphorylation.  Ref: 11438574 J Neurosci, 2001

- :-)

  **Parallel relationship** (e.g. studied together, co-existance, homology, etc.)

  - These findings indicate that  **PKC**  phosphorylates  **tau**  via the MAP kinase pathway, but that PKM can bypass this requirement, therefore demonstrating that distinct intracellular pathways can be mediated by  **PKC**  and PKM.  Ref: 10629766 Brain Res, 1999
  - Expression of  **PKC**  delta, theta, lambda and  **tau**  was not detected in either lymphocytes or ASM.  Ref: 11811627 Indian J Biochem Biophys, 2001
  - We found a mostly combined induction of MDR1, LRP,  **PKC**   **tau**  and  **PKC**  zeta by CAM, ETO and VCR.  Ref: 12553062 Anticancer ResAnticancer Res, 2001
  - We examined the inducibility of drug resistance MDR1, MRP1, LRP and protein kinase C  **PKC**  isozyme alpha, epsilon, eta, theta,  **tau** , zeta corresponding genes in A2780 ovarian cancer cells after a 24 hour treatment with adriamycin ADR, camptothecin CAM, etoposide ETO or vincristine VCR.  Ref: 12553062 Anticancer ResAnticancer Res, 2001
  - It is suggested that fragmentation of  **PKC**  delta during the process of apoptosis results in the phosphorylation and inactivation of GSK 3 and consequently inhibition of the phosphorylation of  **tau** .  Ref: 10708767 FEBS Lett, 2000
  - We analyzed the effects of PKNalpha and protein kinase C  **PKC**  on phosphorylation of  **tau**  protein by glycogen synthase kinase GSK 3beta using monoclonal antibodies AT8, AT180, and AT270.  Ref: 10873588 Biochem Biophys Res Commun, 2000
  - We examined the downstream consequences of  **PKC**  activation by the phorbol ester TPA and by ionophore A23187 mediated calcium influx on phosphorylation of the microtubule associated protein  **tau** .  Ref: 10629766 Brain Res, 1999
  - Our analysis points to a possible link between atypical  **PKC**   **tau**   **PKC**  zeta and MDR1 LRP in cytostatic stress response of cancer cells.  Ref: 12553062 Anticancer ResAnticancer Res, 1999
  - As is the case for the C2 domain, k off for full length  **PKC**  betaII increases when calcium is rapidly removed, reducing  **tau**  from 11 s in the presence of calcium to 48 ms in its absence.  Ref: 11683630 Biochemistry, 2001
  - The individual and sequential influence of protein kinase C  **PKC** , protein kinase A PKA and mitogen activated protein kinase MAP kinase on human brain  **tau**  was examined.  Ref: 10081606 Cell Mol Neurobiol, 1999
  - We identified among these proteins an 8 0 kDa and the  **tau**  protein as specific substrates for protein kinase A PKA, MARCKS, and neurogranin as specific substrates for protein kinase C  **PKC** , and prostaglandin D synthase as substrate for casein kinase II CKII.  Ref: 10226777 Hippocampus, 1999
  - As certain muscarinic receptors are linked to  **PKC** , we examined the effect of a range of agonists on GSK 3beta phosphorylation of  **tau** .  Ref: 11129110 J Neural Transm, 2000
  - Our objective was to test whether bIFN  **tau**  could block PGF 2 alpha production and synthesis of phospholipase A 2 PLA 2 and cyclooxygenase 2 COX 2 enzymes induced by a protein kinase C  **PKC**  stimulator phorbol 1 3 dibutyrate.  Ref: 10906045 Biol Reprod, 2000
  - Prior phosphorylation by  **PKC**  enhanced the nature and extent of AD like  **tau**  antigenicity generated by subsequent incubation with MAP kinase yet inhibited that generated by subsequent incubation with PKA.  Ref: 10081606 Cell Mol Neurobiol, 1999
  - IFN  **tau**  may regulate the PGF 2a synthetic pathway by reducing activity of  **PKC**  or  **PKC**  mediated events.  Ref: 10764976 Domest Anim Endocrinol, 2000
